# Effects of Built Direction and Deformation Temperature on the Grain Refinement of 3D Printed AlSi10Mg Alloy Processed by Equal Channel Angular Pressing (ECAP)

**DOI:** 10.3390/ma16124288

**Published:** 2023-06-09

**Authors:** Przemysław Snopiński, Krzysztof Matus, Ondřej Hilšer, Stanislav Rusz

**Affiliations:** 1 Department of Engineering Materials and Biomaterials, Silesian University of Technology, 18A Konarskiego Street, 44-100 Gliwice, Poland; 2 Materials Research Laboratory, Silesian University of Technology, 18A Konarskiego Street, 44-100 Gliwice, Poland; krzysztof.matus@polsl.pl; 3 Faculty of Mechanical Engineering, VSB-TU Ostrava, 17. listopadu 2172/15, 708 00 Ostrava-Poruba, Czech Republic; ondrej.hilser@vsb.cz (O.H.); stanislav.rusz@vsb.cz (S.R.)

**Keywords:** AlSi10Mg, build direction, ECAP, grain refinement, microstructural characterization

## Abstract

In this work, we used an AlSi10Mg alloy produced by selective laser melting (SLM) to study the effects of build direction and deformation temperature on the grain refinement process. Two different build orientations of 0° and 90° and deformation temperatures of 150 °C and 200 °C were selected to study this effect. Light microscopy, electron backscatter diffraction and transmission electron microscopy were used to investigate the microtexture and microstructural evolution of the laser powder bed fusion (LPBF) billets. Grain boundary maps showed that the proportion of low-angle grain boundaries (LAGBs) dominated in every analysed sample. It was also found that different thermal histories caused by the change in build direction resulted in microstructures with different grain sizes. In addition, EBSD maps revealed heterogeneous microstructures comprising equiaxed fine-grained zones with ≈0.6 μm grain size and coarse-grained zones with ≈10 μm grain size. From the detailed microstructural observations, it was found that the formation of a heterogeneous microstructure is closely related to the increased fraction of melt pool borders. The results presented in this article confirm that the build direction has a significant influence on the microstructure evolution during the ECAP process.

## 1. Introduction

Severe plastic deformation (SPD) is the most widely used method to produce ultrafine-grained materials, which have better mechanical properties compared to their coarse-grained counterparts [1]. In SPD processing, a metal ingot is subjected to extremely high strains [2]. This results in the formation of ultrafine grains and other microstructural components such as nanoprecipitates, nanoclusters, nanotwins, dislocation substructures, etc. [3]. These changes in the microstructure of the material result in improved strength, fatigue resistance and wear resistance, making ultrafine grained (UFG) materials extremely attractive for a variety of industrial applications [4,5].

One of the most popular SPD processes is the Equal Channel Angular Pressing (ECAP). In this process, a metal ingot is pressed through a die with two channels that intersect at an angle (usually 90 or 120 degrees) [6], resulting in significant deformation of the material by simple shear. Extensive research over the past three decades has clearly demonstrated that the excellent strengthening effect of the ECAP process is directly related to the development of an ultrafine grain structure [7,8,9]. These findings have led to considerable interest in the underlying microstructural changes. However, despite the extensive research, understanding the microstructural evolution of LPBF metal alloys under extreme shear deformation is still in its infancy.

Laser powder bed fusion (LPBF) uses a laser beam to selectively melt fine layers of metal powder, enabling the direct fabrication of homogeneous metal objects, layer by layer, from 3D computer-assisted drawing data (CAD) [10]. LPBF technology is widely used in various industries, universities and medical fields. This is due to its advantageous features, including low-cost production, precise dimensional accuracy and customizable geometry, despite its relatively slow production rate. In addition, end products made with LPBF do not require additional manufacturing processes, although sometimes heat treatment or near net-shaping may be necessary [11,12].

One of the noteworthy features of the LPBF alloys is their unique microstructure. It is multi-scale hierarchical and contains ultrafine and metastable microstructures resulted from short laser–material interaction time and high solidification rates in the range of 10^3^–10^8^ K/s [13]. In the case of the AlSi10Mg alloy, two hierarchies can be defined, namely melt pool hierarchy and Al cell hierarchy [14]. At the melt pool hierarchy, the microstructure is composed of the columnar grains that develop due to the epitaxial grain growth along the built direction; whereas, at the Al cell hierarchy, the microstructure is composed of the Si/Al interfaces and sub-micron sized supersaturated Al cells [15,16]. These microstructural features significantly enhance the strength and strain-hardening capability of the additively manufactured (AM) alloys compared with traditional counterparts [17].

It was also found that the build direction affects the thermal history of 3D printed alloys [18]. This impact is explained by the fact that a change in build direction increases the number of molten layers required to build a given object. For example, a cylindrical specimen printed in the horizontal direction requires fewer layers than a vertical-printed counterpart. Consequently, the time period during which the laser beam scans a given layer, and thus the activation of diffusion-driven processes, is determined by the build direction [19]. Moreover, Xiong et al. [20] pointed that the morphology and distribution of the melt pool boundaries varies with the build direction. In addition, Hadadzadeh et al. [21], who studied the microstructure of an AM AlSi10Mg alloy, found that the vertically printed sample contained 75% columnar grains, while the horizontally printed sample contained about 50% columnar grains, and most of them were equiaxed.

Consequently, it can be assumed that manufacturing parameters such as build direction play an important role in the development of an ultrafine-grained microstructure when the additively manufactured sample is plastically deformed.

In this study, the AlSi10Mg specimens were designed with two different build directions (0° and 90° design angles) and then fabricated using the same process parameters by LPBF. Afterwards, the specimens were post-processed via ECAP at two different deformation temperatures. The microstructure was analysed by optical, scanning electron and transmission electron microscopy.

Although there are studies focusing on the effect of SPD post-processing of AM Al-Si alloys [22,23,24], this article is the first which takes into account the effect of design angles in terms of their effect on further grain refinement caused by ECAP processing.

## 2. Methodology

The AlSi10Mg alloy samples were manufactured using an SLM TruPrint 1000 system from Trumpf (Ditzingen, Germany). The following 3D-printing parameters were used to obtain dense samples:

- Laser power (P)—175 W,
- Scanning speed (v)—1.4 m/s,
- Layer thickness (d)—0.02 mm.

The AlSi10Mg alloy samples were fabricated using bi-directional island scanning strategy, and the angle between each adjacent layer was 90°. The chemical composition of the spherical powder supplied by Sigma Aldrich (Steinheim, Germany) is shown in Table 1.

**Figure 1:**
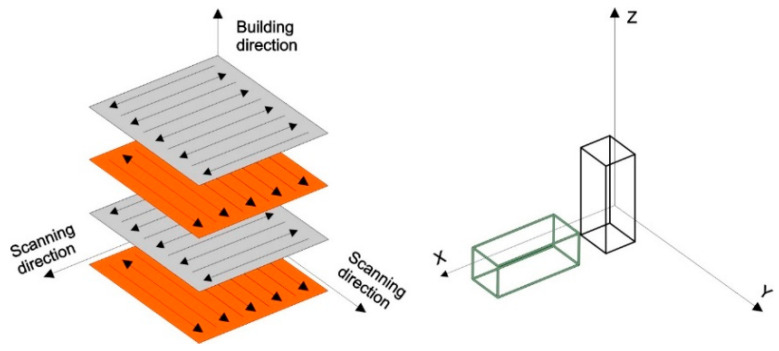
Schematic illustration of the applied bi-directional scanning strategy and sample build directions (black sample—vertically printed, grey sample—horizontally printed).

In this study, we produced two batches of 3D-printed components. The first batch, with dimensions 14.75 × 14.75 × 60 mm, was printed vertically, and the second, with the same dimensions, was printed horizontally, as shown schematically in Figure 1.

After the AM process, the samples were heat treated in a laboratory dryer at 280 °C for 9 min to increase the technological plasticity. Subsequently, the horizontally and vertically printed specimens were subjected to the ECAP process (each sample was subjected to one ECAP pass via route A). SPD process was performed at a press speed of 20 mm/min under two conditions—at 150 °C and 200 °C. A rectangular cross-section die with a channel intersection angle of 90° and a radius of curvature of 0° was used, the design of which was recently reported in this study [25]. This design geometry leads to a true strain (ε) of ~1 per pass, according to the following equation:

(1) $$\varepsilon_{N}=\frac{N}{\sqrt{3}}\left\lfloor 2cot\left(\frac{\varphi }{2}+\frac{\psi }{2}\right)+\psi csc\left(\frac{\varphi }{2}+\frac{\psi }{2}\right)\right\rfloor$$

In this equation, *ε_N_* is equivalent strain after *N* passes; *N* is the number of passes; *ψ* is the angle associated with the arc of curvature; and *φ* is the channel angle.

To reduce the friction between the sample and the die channel, the external surfaces of the work samples were covered with a thin layer of MoS_2_ lubricant.

The microstructures of the samples were examined with an Axio Observer Z1 inverted light microscope (Carl Zeiss NTS GmbH, Oberkochen, Germany) and a Zeiss Supra 35 scanning electron microscope (Carl Zeiss NTS GmbH, Oberkochen, Germany) equipped with an energy dispersive spectrometer (EDS). The crystallographic data of all ECAP processed were characterized by Electron Backscatter Diffraction (EBSD) in the scanning electron microscope operating at 20 KV using a step size of 0.2 μm (heat-treated samples) and 0.12 μm (ECAP-processed samples). Samples for the EBSD analysis were prepared according to the standard metallography procedure comprising grinding with SiC papers and polishing with diamond pastes. The microstructure was revealed by etching the polished surfaces with a Keller’s reagent of which chemical composition was reported in this study [26].

To characterize the microstructures in greater detail, transmission electron microscopy (TEM) specimens were prepared using a focused ion beam (FIB). The TEM lamellae were cut in the ND-TD plane (cross-section) parallel to the ED plane. TEM and high-resolution TEM (HRTEM) were performed using a Titan 80–300, FEI S/TEM microscope equipped with correctors for spherical aberration in both the probe and imaging optics. The microscope was operated at 300 kV.

## 3. Results

**Figure 2:**
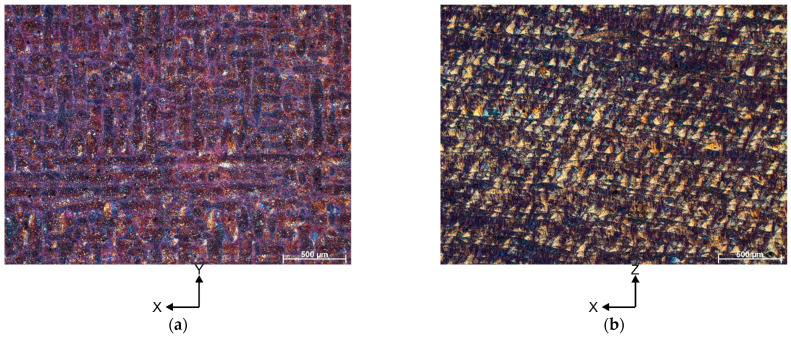
Representative OM microstructures of the AlSi10Mg alloy in a heat-treated condition (**a**) xy plane, (**b**) xz plane (notice that scale bar size is 500 µm).

Figure 2 shows the representative optical microscopic (OM) microstructures of the heat-treated AlSi10Mg alloy, taken in the build-up direction (X-Y) and the scanning direction (X-Z). The OM image of a vertical cross section in Figure 2a shows discontinuous laser scan paths with a 90° rotation between contiguous layers. These scan tracks have a width of about 50–60 µm, and within their interiors, small grains having a diverse crystallographic orientation can be distinguished. In contrast, the OM image of the horizontal cross section (Figure 2b) shows the semi-circular “fish scale” patterns typical of selective laser melting technology. Within these patterns, smaller grains with a columnar morphology can be seen. The different built direction is clearly reflected in the strong orientation of the grains in the built direction (grains are nearly perpendicular to the building direction).

**Figure 3:**
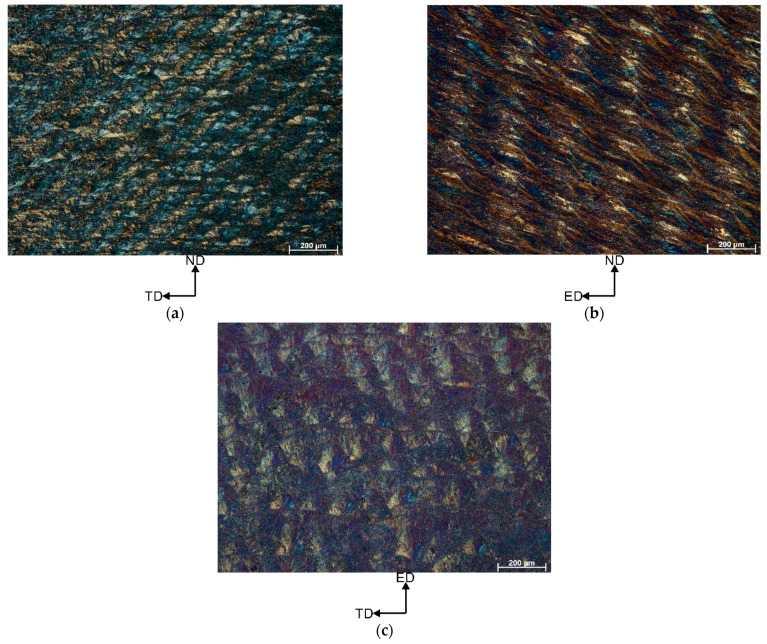
Microstructures of the vertically printed AlSi10Mg alloy subjected to 1 ECAP pass at 150 °C (**a**) ND-TD plane, (**b**) ED-ND plane, (**c**) ED-TD plane (notice that scale bar size is 200 µm).

Figure 3 shows the OM microstructures of the vertically printed AlSi10Mg alloy sample subjected to 1 ECAP pass at 150 °C. The OM image of the ND-TD plane (Figure 3a) shows the semi-circular patterns that replaced the discontinuous scan traces (as a result of the unique shear pattern in the ECAP process). In the ED-ND plane, the shear pattern results in stretching of the microstructure (Figure 3b). As a result, we see a band-like structure inclined at an angle θ~30° to the axis of the exit channel of the die. In contrast, the ED-TD plane microstructure is composed of the overlapping laser scan traces and semi-circular “fish scale” patterns, (Figure 3c). Similar to the starting condition (condition prior to deformation), these patterns contain smaller grains with strong crystallographic texture.

**Figure 4:**
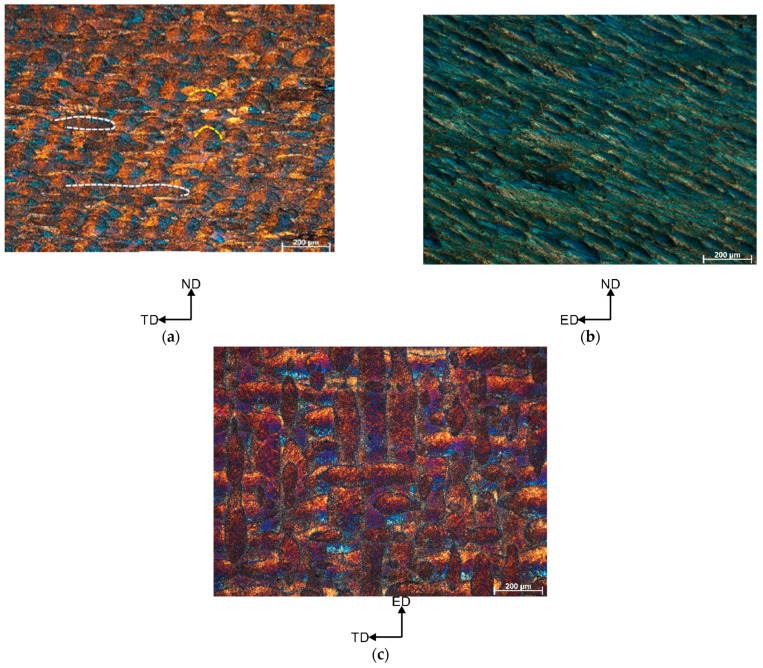
Microstructures of the horizontally printed AlSi10Mg alloy subjected to 1 ECAP pass at 150 °C (**a**) ND-TD plane, (**b**) ED-ND plane, (**c**) ED-TD plane (notice that scale bar size is 200 µm).

Figure 4 shows the microstructures of the horizontally printed AlSi10Mg alloy sample subjected to 1 ECAP pass at 150 °C. As can be seen, the microstructure on the ND-TD plane differs significantly from the vertically printed counterpart. It consists of the discontinuous laser scan traces (see white dotted lines in Figure 4a that separate semi-circular “fish-scale” patterns. The ED-ND plane consists of the elongated and pancaked “fish-scale” patterns inclined at an angle θ~30° to the axis of the exit channel of the die (Figure 4b). What is striking here is that the ED-TD plane remains almost unaffected by the shear pattern, and it comprises of the mutually overlapping discontinuous laser scan traces (Figure 4c).

**Figure 5:**
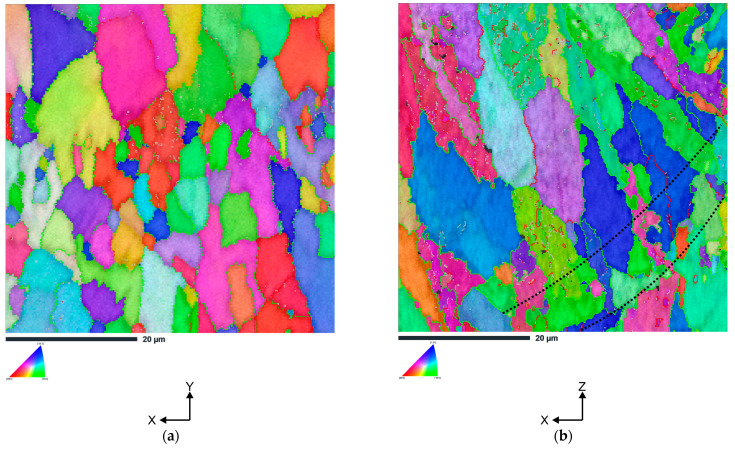
The IPF-Z images of the AlSi10Mg alloy (**a**) vertically printed x-y plane, (**b**) horizontally printed x-z plane (the grey lines denote the low-angle grain boundaries in the range of 2° < θ < 5°, the red lines denote the low-angle grain boundaries in the range of 5° < θ < 15°, and the green lines denote the high-angle grain boundaries in the range of 15° < θ < 65°). (In the case of the x-y plane, the low-angle grain boundaries constitute to about 17%; whereas, in the case of the x-z plane, they constitute to about 24%).

Figure 5 shows the inverse pole figures (IPF) of the AlSi10Mg alloy in the heat-treated condition, focusing on both the cross-sectional view (x-y plane) and the side view (x-z plane). It is evident that the majority of grains in the cross-sectional plane have equiaxed morphology (Figure 5a). In addition, the Al grains in the central region of the laser scan track are significantly larger than the grains that form at the edges of the track. Similar to dendritic structures, a change in building direction from vertical to horizontal results in a change in grain morphology from equiaxed to columnar. From this reason, in the side (x-z) plane, elongated grains with a predominant (111) orientation are observed (Figure 5b). These grains originate at the lower boundary of the molten pool (see black dotted lines) and grow predominantly in the Z-direction when the solidification boundary moves opposite to the direction of heat transfer. A similar observation has already been made by many researchers [27,28,29].

From the EBSD results, we can also summarize that on the cross-sectional view (x-y plane) and the side view (x-y plane), bimodal grain size distribution was revealed (which can be explained by thermal transfer history). Moreover, it is clearly visible that the average grain size on the cross-sectional view (x-y plane) is slightly larger than on the side view (x-z plane)—5.0 µm and 4.3 µm, respectively.

**Figure 6:**
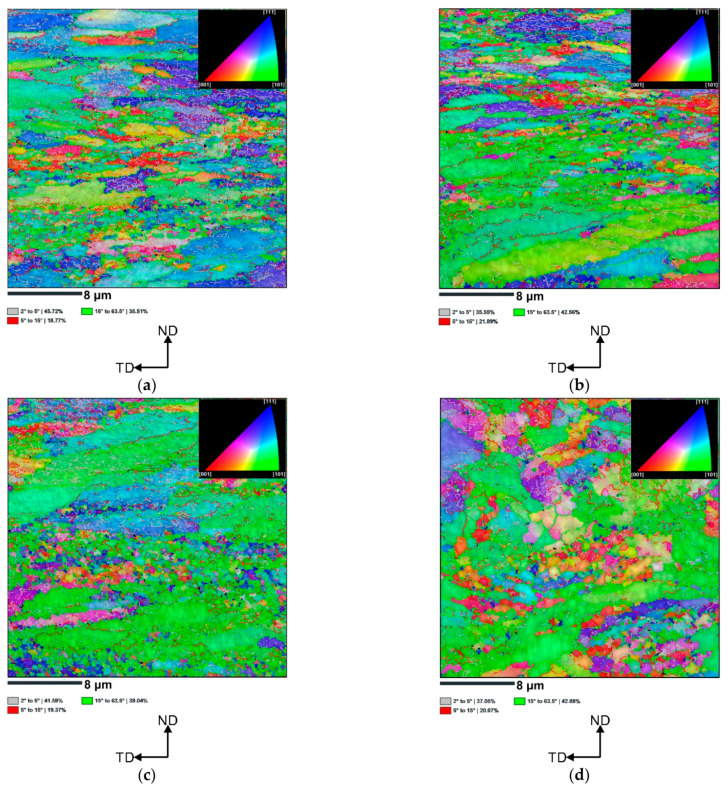
The IPF-Z images of the AlSi10Mg alloy (**a**) vertically printed subjected to 1 ECAP pass at 150 °C, (**b**) vertically printed subjected to 1 ECAP pass at 200 °C, (**c**) horizontally printed subjected to 1 ECAP pass at 150 °C, (**d**) horizontally printed subjected to 1 ECAP pass at 200 °C.

Figure 6 shows the IPF-Z maps of the vertically and horizontally printed AlSi10Mg alloy samples subjected to 1 ECAP pass at 150 °C and 200 °C. As can be seen in Figure 5a, for the vertically printed sample subjected to 1 ECAP pass at 150 °C, the low-angle grain boundaries (LAGBs) account for about 64%. These LAGBs mainly separate elongated grains and smaller subgrains (dislocation cells) located inside. On the other hand, ultrafine grains are mainly separated by high-angle grain boundaries (HAGBs). This indicates that dynamic recrystallization (DRX) occurred during ECAP processing. As the deformation temperature increases, the fraction of ultrafine grains decreases significantly (Figure 6b). Moreover, the fraction of LAGBs decreases slightly to about 57%, indicating that some LAGBs transform into HAGBs. Similar to the previous sample, the LAGBs separate large, elongated grains, while the HAGBs separate equiaxed DRX grains.

A comparable microstructure was observed for the horizontally printed AlSi10Mg samples subjected to 1 ECAP pass at 150 °C and 200 °C. One can observe that the initial coarse grains are subdivided by rather smooth dislocation boundaries with low-to-moderate angle misorientations. From the statistically large analysis using EBSD, it is clear that for the horizontally printed sample subjected to 1 ECAP pass at 150 °C, the low-angle grain boundaries (LAGBs) account for about 61%; whereas, for the specimen deformed at 200 ºC (LAGBs), they account for about 57% (Figure 6c,d), which similarly can be ascribed to the DRX process (increased nucleation rate of DRX nuclei at higher temperature).

The reported enormous grain refinement can be related to the ECAP process’s simple shear deformation, which leads to a particularly large slip concentration in a 111-plane. The Al/Si interfaces as well as the pre-existing dislocation networks [30] in the investigated SLM-AlSi10Mg alloy restrict the dislocation slip, resulting in a very rapid accumulation of dislocations around heterogeneous interfaces [31,32], which accelerates the refinement of cell blocks (after the ECAP process, new subgrains are formed at pre-existing grain boundaries). Consequently, using EBSD, we observed a microstructure in which large grains are composed of small subgrains (with low misorientation angle), and these subgrains correspond to the one cell boundary.

**Figure 7:**
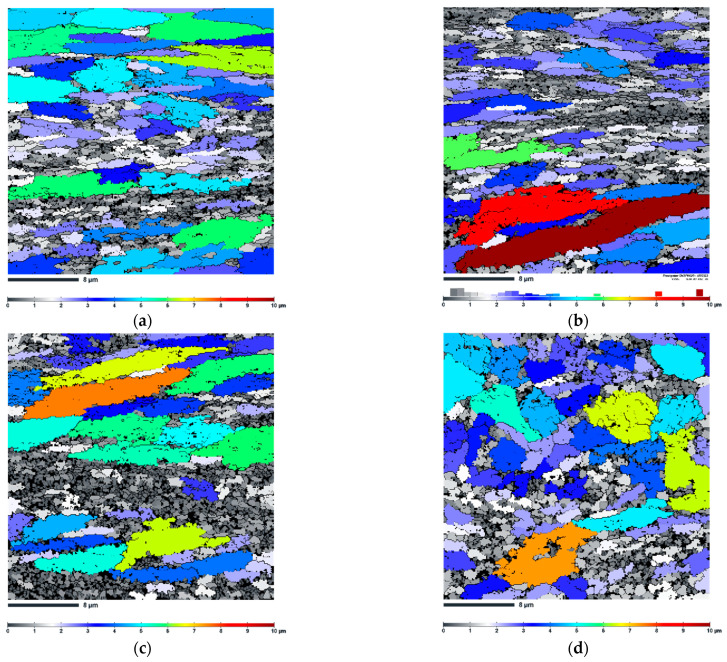
The grain size distribution maps of the AlSi10Mg alloy (**a**) vertically printed subjected to 1 ECAP pass at 150 °C, (**b**) vertically printed subjected to 1 ECAP pass at 200 °C, (**c**) horizontally printed subjected to 1 ECAP pass at 150 °C, (**d**) horizontally printed subjected to 1 ECAP pass at 200 °C (notice that scale bar size is 8 µm).

Figure 7 shows the grain size maps of the vertically and horizontally printed AlSi10Mg alloy samples subjected to 1 ECAP pass at 150 °C and 200 °C. The average grain size calculated by the EBSD software with GTA = 5° is summarised in Table 2. It can be clearly seen that a heterogeneous grain size distribution was formed in the four samples studied, including both coarse-grained and fine-grained regions. Referring to IPF-Z EBSD maps (Figure 6), the coarse-grained regions correspond to the interior of the melt pool, where columnar grains are typically present. These columnar grains transform into arrays of planar and extended boundaries as a result of the strong strain accumulation and shear pattern during ECAP processing. On the other hand, the ultrafine grain regions correspond to the melt pool boundaries (MPBs). From the optical microscopy images (Figure 3a and Figure 4a), it can be concluded that the vertically printed ECAP-processed sample contains more MPBs than the horizontally printed ECAP-processed counterpart. This results in more pronounced grain refinement of the vertically printed samples. It is noteworthy to add that temperature effects are also captured by EBSD. In both analysed conditions (horizontal and vertical), the samples deformed at 200 °C have larger grain sizes than those deformed at 150 °C.

**Figure 8:**
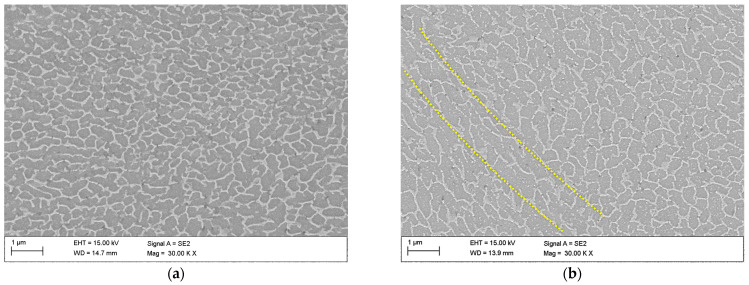
SEM images of vertically printed AlSi10Mg sample deformed at (**a**) 150 °C and (**b**) 200 °C (notice that scale bar size is 1 µm).

To analyse the effects of deformation temperature on the cell morphology of the Al/Si interface, SEM observations were made. Figure 8a,b show the secondary electron micrographs of the vertically printed AlSi10Mg samples deformed at 150 °C and 200 °C, respectively. They show the region near the molten pool boundary (see yellow dotted lines). As can be seen, partial decay and spheroidization of the eutectic Si network occurred in both samples. From the microstructures comparison, it can also be concluded that the higher deformation temperature promotes the globularization of the Si network, since the sample processed at 200 °C has a thicker Si network. It is hypothesized that keeping at high temperatures causes increased diffusion, which results in spheroidization processes in the Si network and precipitation of supersaturated Si atoms within the cells. This idea is consistent with the findings of Li et al. [33], who investigated the precipitation of Si atoms in the form of nano- or micrometric particles from supersaturated solid solutions in an SLM-processed AlSi10Mg alloy.

**Figure 9:**
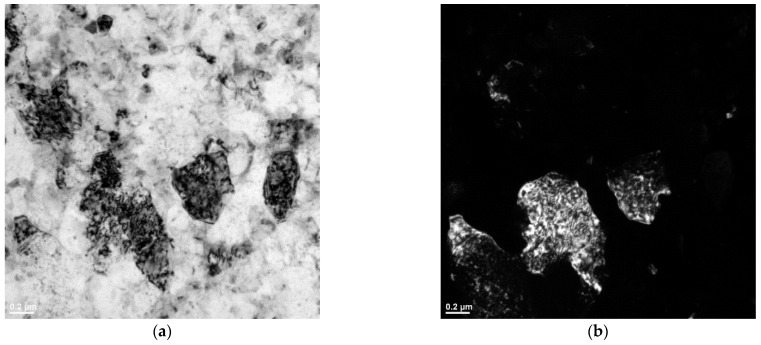
(**a**) Bright- and (**b**) dark-field TEM images of vertically printed AlSi10Mg sample deformed at 150 °C.

**Figure 10:**
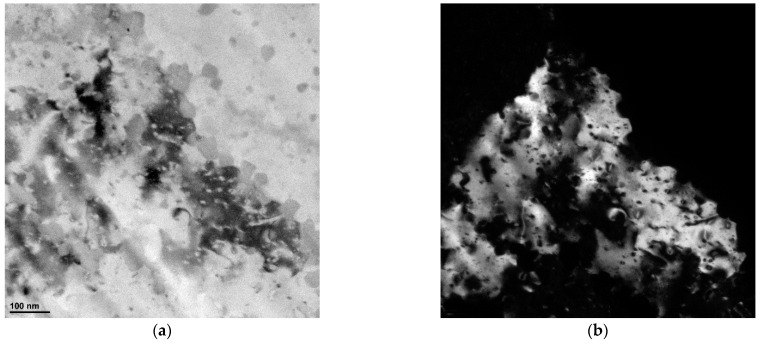
(**a**) Bright- and (**b**) dark-field TEM images of vertically printed AlSi10Mg sample deformed at 200 °C.

Figure 9 and Figure 10 show the results of TEM characterization of the vertically printed AlSi10Mg alloy deformed at 150 °C and 200 °C, respectively. It can be clearly seen that the plastic deformation causes dislocation motion [34]. The TEM micrographs provide clear evidence of massive dislocation activity and its accumulation. Moreover, from the direct comparison of Figure 9 and Figure 10, it can be concluded that more statistically stored dislocations (SSDs) accumulated in the sample deformed at 150 °C. These images also show a large difference in subgrain size between the two samples. For the vertically printed AlSi10Mg samples deformed at 150 °C, subgrains with a size in the range of 400–600 nm were found, while for the sample deformed at 200 °C, a large grain (with a size of about 1 µm) was found in the investigated area. This grain is almost dislocation-free, indicating that dynamic recovery (DRV) and DRX acted simultaneously during ECAP processing at higher temperature.

**Figure 11:**
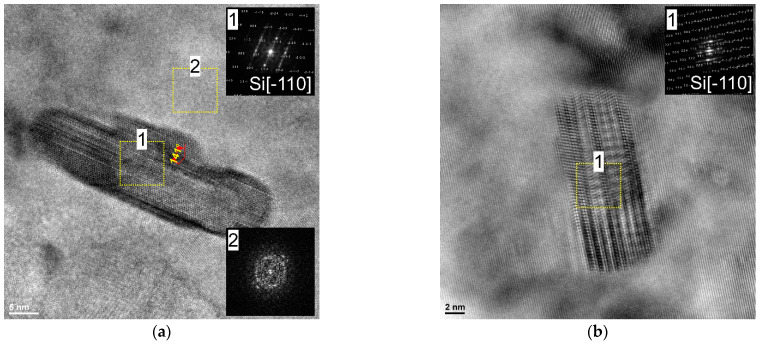
HRTEM images taken at the Al/Si interface (**a**) vertically printed AlSi10Mg sample deformed at 150 °C, (**b**) vertically printed AlSi10Mg sample deformed at 200 °C.

Figure 11 shows the high-resolution TEM (HRTEM) images taken close to the Al/Si interface of the vertically printed specimens deformed at 150 °C and 200 °C. As can be seen, in the vicinity of the Si precipitates, twins/stacking fault exists. Such deformation twins, observed in nanocrystalline materials with a 141° kink boundary, result from the multiplication of partials having identical Burgers vectors.

According to the previously published work [35], at a lower deformation temperature (100 °C), the Si phase exists in the form of amorphous islands. Here it is found that, when the sample is deformed at 200 °C, the Si phase is crystalline and heavily twinned, indicating that the predominant deformation mode of the Si phase is mechanical twinning, while at lower deformation temperature of 150 °C, the silicon is in the crystalline and amorphous form (as shown by the fast Fourier transform (FFT)). This indicates the deformation-induced amorphization of Si phase [36].

## 4. Discussion

In this article, we have investigated the effect of the build direction on the microstructure evolution and grain refinement of LPBF AlSi10Mg alloy specimens subjected to ECAP processing. Comprehensive microstructural investigation revealed that unique and distinct microstructural patterns were formed in the ECAP-processed LPBF samples. Additionally, EBSD study revealed a similar degree of grain refinement regardless of the sample build direction. However, to understand these microstructural changes, it is essential to analyse the metal flow and theoretical shear patterns that occur during the ECAP process. As mentioned in these studies [5,30,37], during the billet’s passage through the deformation zone within the ECAP die, the front part undergoes rotational movement. Subsequently, as the billet traverses the transition region between two die channels, a significant shear force is applied, typically at a 45° angle relative to the top and bottom surfaces of the sample. This shear force often manifests itself in the formation of a layered grain structure. Notably, the EBSD analysis revealed the formation of the layered (pancaked) grains (Figure 6) which is consistent with the theoretical assumptions. However, in each analysed sample, we discovered, additionally, the formation of the bimodal microstructure (pancaked grains coexist with ultrafine grains). As shown in Figure 3, the ND-TD plane exhibited a sequence of semi-circular patterns resembling fish scales, observed in the x-z plane of LPBF specimen, (Figure 2b). These patterns differed from the arrangement of discontinuous laser scan traces, (Figure 2a), observed prior to deformation. It can be concluded that this transformation of microstructure resulted from the initial rotation of the front section of the billet, which has been followed by significant shear forces (in the channel intersection area) that altered grain shapes. As shown above, this phenomenon caused a decrease in the distance between the heat-affected zones (HAZ) due to greater deformation within the HAZ compared to the interior of the melt pool during ECAP processing. The spherical Si particles present in the HAZ contributed to a weakened load transfer effect, resulting in a lower macro yield point. As a consequence, localized plastic deformation predominantly occurred in the HAZ region. Therefore, a bimodal grain structure was obtained in which elongated and ultrafine grains exist together.

A similar evolution of the microstructure was observed in the horizontally printed specimen. The x-z plane originally consisted of several “fish scale” patterns. As a result of billet rotation, these unique pattern arrangements in the ND-DT plane changed—it consists of discontinuous laser scan traces (see white dotted lines in Figure 4a) which separate semi-circular “fish scale” patterns (yellow dotted lines). In addition, similar to the vertically printed sample, the distance between the heat-affected zones (HAZ) decreased. This resulted in the formation of a bimodal microstructure in which layered (pancaked) grains correspond to the scan tracks and fish scale patterns interiors, while ultrafine grains correspond to the HAZ zones. Moreover, such unique microstructure evolution resulted in different degrees of grain refinement for both vertically and horizontally printed samples. This can be caused by different proportion of HAZ zones in the investigated plane. Clearly, the ECAP-processed, vertically printed sample on the ND-TD plane had more HAZs consisting of ultrafine grains. This resulted in a more pronounced grain refinement.

This finding has important implications for the application of AlSi10Mg alloys in various industries. The ability to control microstructural evolution through printing direction and subsequent ECAP processing opens up new opportunities to improve the mechanical properties and performance of additively manufactured components. This work suggests that by strategically selecting the printing direction and applying appropriate post-processing techniques such as ECAP, it is possible to tailor the microstructure of AlSi10Mg alloy for specific applications. In addition, this study serves as a stimulus for future work investigating the effects of printing direction and post-processing techniques on other alloy systems, expanding our understanding of additive manufacturing and its potential for advanced materials engineering.

The structural analyses performed in this study via TEM revealed the presence of crystalline Si embedded in an amorphous layer in an AlSi10Mg sample deformed at 150 ºC. In contrast, the sample deformed at 200 °C contained only Si in crystalline form. This indicates that lower deformation temperatures inhibit the long-distance migration of elemental point defects generated by ECAP processing. Consequently, at higher deformation temperatures, a greater accumulation of defects may be required to initiate a direct transformation from the crystalline state to the amorphous state. Therefore, in the AlSi10Mg sample deformed at 200 °C, the Si phase is in crystalline form. On the other hand, if the sample is deformed at a lower temperature (150 °C), there is a higher accumulation of dislocations at the Al/Si interface, resulting in a larger step and subsequent stress concentration. Consequently, this higher defect accumulation can directly lead to the phase transformation Si-I—>a-Si (crystalline Si—>amorphous Si).

## 5. Conclusions

In this paper, we studied the effect of build direction and deformation temperature on the mechanism of grain refinement of 3D-printed AlSi10Mg aluminium alloy. The main conclusions from this study can be summarized as follows:

- Optical microscopy showed a unique evolution of the microstructure of the samples studied. It was found that depending on the build direction, the proportion of the molten pool boundaries in the cross-sectional plane changed.
- In each condition studied, the microstructure was inhomogeneous. The coarse and fine grains coexist in the sample, and many fine DRX grains were distributed around the molten pool boundaries.
- An ultrafine microstructure is achieved after ECAP processing of LPBF AlSi10Mg samples. The average grain size was refined from ~5 μm to ~1 μm.
- The samples processed with ECAP contained a very high density of LAGBs. DRX-HAGBs were found to form around the edges of the molten pool boundaries.
- Samples deformed at lower temperatures had finer equiaxed grains with a lower proportion of high-angle boundaries, indicating the formation of a substructure.
- TEM revealed that the Si phase is deformed mainly by mechanical twinning at the two deformation temperatures investigated.

## Figures and Tables

**Table 1:** Chemical composition of the AlSi10Mg alloy powder.

Si	Mg	Ti	Cu	Fe	Al
10.5	0.5	0.15	0.15	0.09	Balance

**Table 2:** Grain sizes of the analysed samples (in the grain size analysis, the grains smaller than four pixels were omitted).

SAMPLE	Grain Size, µm	LAGBs, %	HAGBs, %
Vertical ECAP 150 °C	0.84	64.5	35.5
Vertical ECAP 200 °C	0.94	57.5	42.5
Horizontal ECAP 150 °C	1.08	61.0	39.0
Horizontal ECAP 200 °C	1.14	57.0	43.0

## Data Availability

Data available on request.
